# Otitis Media in Children with Severe Acute Malnutrition: A Scoping Review

**DOI:** 10.3390/children12040397

**Published:** 2025-03-21

**Authors:** Casey Jane Eslick, Samantha Govender, Senamile Ntuli, Beleza Rikhotso, Lufuno Zwivhuya Mabada, Selaelo Matjena

**Affiliations:** Department of Speech-Language Pathology and Audiology, School of Health Care Sciences, Sefako Makgatho Health Sciences University, Molotlegi St., Ga-Rankuwa Zone 1, Pretoria 0208, South Africa; samantha.govender@smu.ac.za (S.G.); senamile.ntuli@smu.ac.za (S.N.);

**Keywords:** children, health promotion, middle ear infection, otitis media, severe acute malnutrition, undernutrition

## Abstract

**Background**: Severe acute malnutrition (SAM) increases susceptibility to infections, including otitis media (OM). Research establishes the association between malnutrition and OM. **Objectives**: This scoping review specifically explored the prevalence, risk factors, co-morbidities, and management strategies for OM in children under 5 years with SAM with the goal of identifying future research directions to address gaps in the literature. **Methods**: A scoping review methodology was adopted to identify the English literature published since 2014. The Arskey and O’Malley framework and Preferred Reporting Items for Systematic Reviews and Meta-Analyses Extension for Scoping Reviews (PRISMA-ScR) guidelines were followed. EBSCOhost, PubMed, Medline, Scopus, Science Direct, and Google Scholar were searched using keywords to refine the search. **Results**: Seven papers met the inclusion criteria, showing limited studies were available on the topic. Four deductive themes, including prevalence, risk factors, co-morbidities and management strategies, were explored. None of the studies suggested the role of an audiologist in SAM management. **Conclusions**: Children with SAM face an increased risk of OM due to multiple factors. Associated hearing loss underscores the importance of community-based interventions. Interprofessional collaboration, community education, and integrating audiologists can enhance early OM detection and management for children with SAM. Prospective research and education on audiologists’ roles in SAM management can promote health outcomes in this vulnerable population.

## 1. Introduction

Severe acute malnutrition (SAM) is a serious nutritional condition that affects approximately 13.7 million children under five years worldwide, predominantly in low- and middle-income regions [1]. Deficits in essential nutrients lead to severe weight loss, muscle wasting, and poor health outcomes in children with SAM [2]. SAM is diagnosed in children with a weight-for-height measurement of more than three standard deviations (70%) below the median WHO growth standards or by the presence of nutritional oedema [2]. SAM places children at high risk of mortality, impairs immune function, and increases their susceptibility to infections, such as otitis media (OM) [3,4]. Among comorbidities associated with SAM, OM is often under-recognised.

OM is defined as inflammation or infection of the middle ear, commonly affecting up to 75% of young children by age three [5,6]. The various types of OM contribute to morbidity, speech delays, and long-term developmental implications [5,6,7]. Acute otitis media (AOM), chronic otitis media (COM), and chronic suppurative otitis media (CSOM) vary in aetiology, severity, recurrence, and health implications [8,9]. AOM involves the rapid onset of infection, whereas COM involves recurrent, persistent inflammation often due to inadequate treatment [10,11]. CSOM can lead to structural changes in the auditory system, hearing loss, and developmental delays [12,13,14]. Audiologists have a role to manage OM through early identification, hearing assessments, interprofessional collaboration, and strategies to mitigate long-term impacts of hearing loss [9].

Research establishes that malnourished children are vulnerable to auditory pathology, particularly OM [11,15,16,17]. Biological and environmental factors are recognised contributors to OM and are also notable risk factors for the development of SAM in young children [5,8,10,18,19]. Biological risk factors include prematurity, eustachian tube dysfunction, frequent colds or flu, and allergies, and environmental factors include low socio-economic status and poor feeding practices [5,8,10,18,19]. Reviews show similar biological and environmental risks for SAM and OM that increase susceptibility to infections, including food insecurity, overcrowding, adverse living conditions, and inadequate healthcare access [8,12,15,19,20]. For children with SAM, nutritional deficiencies compromise immunity and mucosal barriers, increasing the risk of OM infection [4,21]. This review aims to collate risk factors for children with SAM that can predispose them to OM to support a more targeted approach to managing cases of OM in children with SAM.

Comorbidities such as HIV and TB exacerbate immune dysfunction and can impact the severity and recurrence of SAM and OM [14,22]. In developed contexts, advanced healthcare infrastructure reduces the burden of SAM and OM. However, in resource-limited settings where healthcare is constrained, co-morbidities, and infections occur frequently, complicating intervention [20]. Understanding the co-morbidities in children with OM and SAM is important for informing clinical interventions. Exploring this gap can provide insight into the burden of disease for this vulnerable population. Delayed intervention can contribute to speech, cognitive, and developmental delays with long-term educational disadvantages [7,9,23,24,25].

SAM is associated with developmental delays across multiple domains and educational disadvantages [23,24]. One-year-old children with SAM are shown to experience gross motor delays up to 300% and 71.4% in language skills [23]. Further research shows 61.1% of children with SAM presented with global developmental delays [24]. Untreated OM and subsequent hearing loss are associated with reduced cognitive functions [7]. Limited language skills, lower IQ, poor attention, and slower processing speed contribute to low cognitive scores and poor school achievement in adolescence [7].

Existing scoping and systematic reviews investigate SAM and OM separately, focusing on prevention, nutritional rehabilitation, treatment protocols, reducing mortality or relapse, and infection control in the paediatric population. Systematic reviews by Elemraid et al. [15] and Salamah et al. [17] explored micronutrient deficiencies and the pathogenesis of OM, indicating nutritional supplementation can effectively prevent and treat OM. However, reviews on OM lack clear nutritional diagnoses and do not specify SAM. Research reflects various co-morbidities associated with SAM; however, when OM is described, it is often grouped with other co-morbidities with little detailed analysis [8,26]. Despite understanding how OM and SAM could impact development independently, the intersection of SAM and OM is not well described. It is unclear how immune changes related to SAM may impact the risk of developing OM, recurrence, or severity. The interaction of SAM and the onset, severity, and recurrence of the various types of OM is underexplored. Furthermore, clinical management strategies for OM in children with SAM are inadequately documented.

Despite the recognised relation between malnutrition and OM, gaps in the literature persist, warranting further exploration to guide healthcare policies and interventions [17]. Therefore, this scoping review is needed to explore the prevalence of OM in children with SAM and identify risk factors, common co-morbidities, and management strategies specifically for this population. This review aims to comprehensively synthesise available evidence to guide health professionals and future research to improve health outcomes for children with SAM at risk of or currently experiencing OM.

## 2. Materials and Methods

A scoping review methodology was used to explore the available literature and enable integrative reporting [27]. The Arskey and O’Malley [28] framework was conducted with recommendations from Levac et al. [29] to inform the procedural steps: (a) identifying the research question, (b) collecting publications, (c) selecting studies, (d) charting data, and (e) collating, summarising, and reporting findings. The researchers complied with the Preferred Reporting Items for Systematic Reviews and Meta-Analyses Extension for Scoping Reviews (PRISMA-ScR) guidelines, which informed decision-making, data extraction, and reporting [30]. This scoping review protocol was registered with Open Science Framework at https://doi.org/10.17605/OSF.IO/TM6RD.

### 2.1. Research Question

The current review was guided by the following questions:

- What are the prevalence, risk factors, co-morbidities, and management strategies for OM in children under five years with SAM?
- What future research is needed to address gaps in the literature identified?

### 2.2. Inclusion Criteria

The population in this review was children under five years old, diagnosed with SAM according to the WHO diagnostic criteria [2]. Children under five are a priority for WHO malnutrition interventions, and this age range included the period when children are vulnerable to malnutrition and infections [14]. The health condition included all types of otitis media (AOM, otitis media with effusion (OME), CSOM) to allow a comprehensive understanding of the condition in children with SAM. The concepts explored included prevalence, risk factors, co-morbidities, and management strategies to better understand OM in children with SAM. The context of this review was not limited geographically and included all global publications from diverse healthcare settings. The review focused on the literature published from 2014 to October 2024. This timeframe captures the most recent research and evidence-based practices. Furthermore, updated WHO diagnostic criteria for SAM in children aged 6–59 months were introduced in 2013 [2].

### 2.3. Data Sources and Study Selection

The database search was conducted in October 2024 across EBSCOhost, PubMed, Medline, Scopus, and Science Direct for the published literature and Google Scholar for the grey literature. The search terms (Table 1) related to nutritional status (SAM), health condition (OM), and population (children under 5 years) and were used with Boolean operators (Table 2) for a comprehensive search strategy with breadth of literature [29]. Filters for publication range (2014–present), English language, and full text were applied. The full search strategy per database is available in Table S1.

Duplicates were removed. Study selection continued in two phases. To prevent bias, in phase one, three reviewers independently screened the citation titles and abstracts according to post hoc eligibility criteria as follows [29]: Studies relevant to the research question, regarding OM and nutritional deficiencies, published from 2014 to October 2024 with full-text availability were included. Due to limited resources for translation, only studies in English were included. Studies outside of the scope, including children older than 5 years, not specifying the age range, published outside of the date range, or not available in English full-text were excluded. In phase two, to improve consistency, four reviewers independently screened the remaining full-text publications for inclusion. A fifth reviewer determined the final selection in the case of disagreements. Reference lists of included studies were independently screened for missed literature. Inter-rater agreement was calculated and determined to be 82.43%.

### 2.4. Charting the Data

Two authors independently extracted the data and then combined the findings, which a third author reviewed. Thereafter, researchers continued the analysis.

### 2.5. Summary and Synthesis

A descriptive-analytical approach with deductive analysis was employed to synthesize the findings.

### 2.6. Ethical Considerations

This review upheld ethical standards for research without contact with living subjects. Full ethical clearance for the review was obtained (SMUREC/H/325/2024:UG; Date: 8 August 2024).

## 3. Results

**Figure 1:**
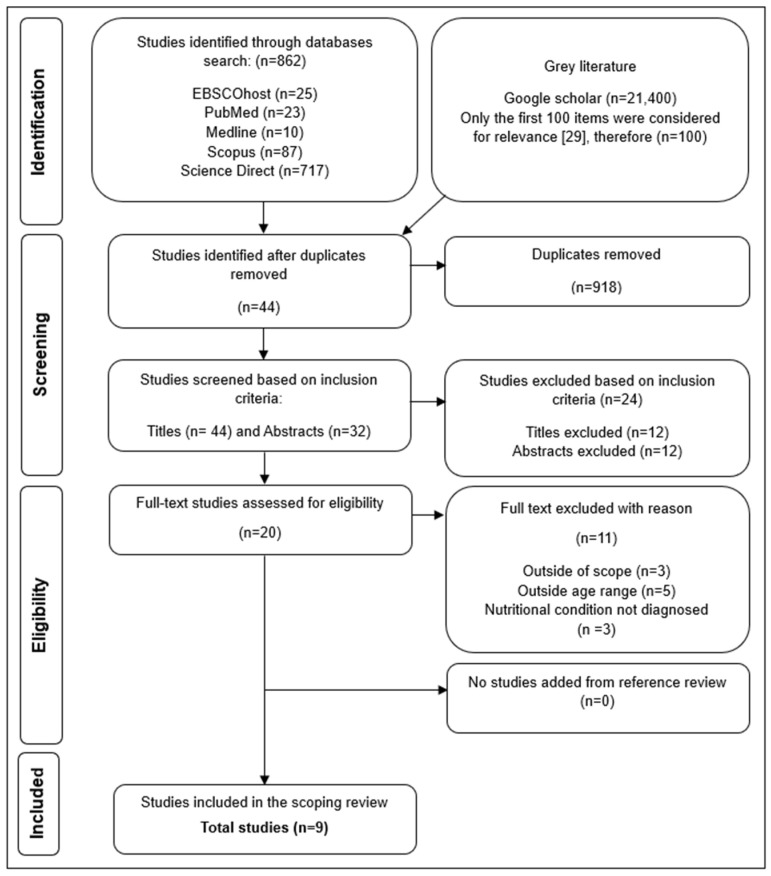
The PRISMA flow diagram depicting the process of study selection [29].

The search identified 962 studies. After duplicates were removed, 44 studies remained. Following title and abstract screening, 24 studies that did not meet the inclusion criteria were excluded. Twenty studies were assessed for eligibility, with 11 excluded for various reasons, as shown in the PRISMA-ScR flow diagram [30] (Figure 1). No additional studies were identified through reference list screening. A total of nine studies met the inclusion criteria and were included in the review.

### 3.1. Study Characteristics

The characteristics of each study are summarised in Table 3. The studies were conducted in low- and middle-income countries and developing contexts. Six out of nine studies occurred in India. Most studies were hospital-based. One study [3] was community-based, and another was based at health clinics [14].

The identified studies predominantly used prospective, cross-sectional, observational, or descriptive designs. One study [31] used a case-control approach. The age range in the majority of studies was 6 months to 5 years. Within this age range, the majority of participants were between 6 and 24 months old [14,24,26,31,32,33,34]. Four studies indicated the majority of participants were from low socio-economic settings [24,26,32,34]. The most recent WHO diagnostic criteria for SAM [2] were used to categorise nutritional status in the majority of studies [3,6,24,26,32,33,34]. One study diagnosed MAM [14], according to WHO criteria. One study used the Welcomes Classification to diagnose protein-energy malnutrition [31]. Studies showed large variations in the type of OM measured. Therefore, the type of OM per study is indicated in Table 4. Findings of the deductive analysis conducted for this scoping review are summarised in Table 4, according to the four themes: prevalence, risk factors, co-morbidities, and management strategies. Each theme highlights key insights into the relationship between OM and children with SAM from the included studies.

**Table 3:** Study characteristics.

Author/s and Year	Study Title	Design	Population	Sample Size	Country	Context
Chaurasiya, Pathak, Gupta, and Chhabra 2018 [32]	Clinical profile of severe acute malnutrition among children under five years of age living in Bundelkhand region of Uttar Pradesh	Prospective, Observational, Cross-sectional	Children aged 1 month to 5 years with SAM	152	India	Developing
Das, K., Das, S., Mohapatra, Swain, and Mohakud 2021 [24]	Risk and adverse outcome factors of severe acute malnutrition in children: A hospital-based study in Odisha	Prospective, Observational, Cross-sectional	Children aged 1 month to 5 years with SAM	198	India	Developing
Das, Paul, Bhattacharya, Basu, Chatterjee, Sen, and Bhakta 2017 [26]	Clinicoepidemiological profile, risk factors and outcome of severe acute malnutrition children at the nutritional rehabilitation centre of a tertiary care centre in Eastern India—A 4 year experience	Prospective, Observational, Cross-sectional	Children aged 6 months to 5 years with SAM	630	India	Developing
Hounkpatin et al., 2016 [3]	Risk factors for acute otitis media in children aged 0 to 5 years in Parakou	Prospective, Descriptive, Cross-sectional	Children aged 0 to 5 years with and without SAM	2040	Benin	Developing
Kamel, Deraz, Elkabarity, and Ahmed 2016 [31]	Protein energy malnutrition associates with different types of hearing impairments in toddlers: Anemia increases cochlear dysfunction	Prospective, Descriptive, Case-control, Cross-sectional	Toddlers aged 6 to 24 months with and without moderate/severe PEM	100	Egypt	Developing
Sahu, Pradhan, Gudu, Tripathy, and Jena 2024 [33]	Prevalence of acute bacterial infections and their antibiotic sensitivity pattern in children with severe acute malnutrition from a tertiary care hospital of Odisha	Prospective, Observational, Cross-sectional	Children aged 6 months to 5 years with SAM	95	India	Developing
Saxena, Bhargava, Srivastava, S., and Srivastava, M. 2016 [6]	Malnutrition among children having otitis media: A hospital based cross-sectional study in Lucknow district	Prospective, Descriptive, Cross-sectional	Children aged 1 month to 5 years with symptoms of ear problems	851	India	Developing
Shalini, and Vidya 2021 [34]	A study on the clinicosocial profile of severe acute malnutrition cases admitted to nutritional rehabilitation centre, Davanagere, Karnataka	Retrospective, Descriptive, Cross-sectional	Children aged 0 to 5 years with SAM	155	India	Developing
Udoh et al., 2024 [14]	Morbidity pattern of under-fives with moderate acute malnutrition in southern Nigeria	Prospective, Descriptive, Cross-sectional	Children aged 6 months to 5 years with MAM	162	Nigeria	Developing

Abbreviations: MAM, moderate acute malnutrition; PEM, protein-energy malnutrition; SAM, severe acute malnutrition.

**Table 4:** Results presented by deductive analysis.

Study	Prevalence of OM	Risk Factors	Comorbidities (with SAM and OM)	Management Strategies
Chaurasiya, Pathak, Gupta, and Chhabra 2018 [32]	0.65% (CSOM)	Low socio-economic conditions, poor feeding practices, limited exclusive breastfeeding, nutritional deficiencies, repeated infections	Anaemia, lower respiratory tract infection, pneumonia, diarrhoea, gastroenteritis, septicaemia, skin/hair changes, malaria, and TB	The study highlighted the importance of screening weight-for-height on children’s presentation for early identification. Nutritional care, particularly using the suckling supplemental technique, and counselling showed improved outcomes in this population.
Das, K., Das, S., Mohapatra, Swain, and Mohakud, 2021 [24]	2.5% (OM type unspecified)	Low socio-economic conditions, lack of safe drinking water, low immunisations, poor feeding practices, limited exclusive breastfeeding	Anaemia, acute respiratory tract infection, gastroenteritis, diarrhoea, septicaemia, urinary tract infection, skin/hair changes, measles, malaria, and TB	To reduce undernutrition and mortality among children with SAM, universal health coverage and immunisation is important. Healthcare facilities should implement routine screening for early identification of SAM.
Das, Paul, Bhattacharya, Basu, Chatterjee, Sen, and Bhakta 2017 [26]	0.79% (OM type unspecified)	Low socio-economic conditions, low immunisations, poor feeding practices, limited exclusive breastfeeding	Anaemia, acute respiratory tract infection, gastroenteritis, diarrhoea, septicaemia, skin/hair changes, measles, malaria, HIV, and developmental delay	The study highlighted that healthcare facilities are necessary for critical nutritional care. Due to the multifactorial nature of SAM, community-based management should be integrated to effectively address causes and sustain recovery.
Hounkpatin et al., 2016 [3]	16.3% (SAM in OM)	Low socio-economic conditions, smoke exposure, nutritional deficiencies, chronic rhinitis, family or personal history of OM	Chronic rhinitis	The importance of early risk factor identification for prevention measures is highlighted. Family education about environmental risks to modify exposure to risks is noted. The audiologist’s role is not mentioned.
Kamel, Deraz, Elkabarity, and Ahmed 2016 [31]	84.6% (OM type unspecified)	Low socio-economic conditions, low parental education, nutritional deficiencies, anaemia	Hearing loss secondary to OM associated with PEM	Monitoring for early detection of auditory pathology may be conducted with neuro-physiological methods, especially in high-risk toddlers.
Sahu, Pradhan, Gudu, Tripathy, and Jena 2024 [33]	2.1% (CSOM)	HIV, TB	Respiratory tract infection, acute gastroenteritis, bacteraemia, urinary tract infection, skin changes, meningitis, measles, malaria, TB, HIV, and ear discharge	The importance of antimicrobial therapy to manage infections and improve clinical outcomes in children with SAM is highlighted. The audiologist’s role is not mentioned.
Saxena, Bhargava, Srivastava, S., and Srivastava, M. 2016 [6]	59.9% (malnourished in OM)	Poor hygiene, food insecurity limited breastfeeding, smoke exposure, nutritional deficiencies	Respiratory tract infection, meningitis, mastoiditis, and sequelae such as hearing loss	A strong, positive correlation between OM and malnutrition is established. The findings recommend that children with stunting or SAM are more prone to severe and repeated OM. There is a need for education on risk factors and ear care for early identification and prevention. An interdisciplinary approach with ENT and paediatrics is promoted. The audiologist’s role is not mentioned.
Shalini, and Vidya 2021 [34]	3.1% (OM type unspecified)	Low socio-economic conditions, lower education level of parents, maternal unemployment, recurrent illness	Anaemia, acute respiratory tract infection, gastroenteritis, diarrhoea, skin/hair changes, TB, congenital heart disease, developmental delay, ear discharge, and skin tags on the ear lobe	The multifactorial nature of SAM is highlighted. There is a need for parent health education about feeding, nutrition, prevention, and health monitoring. Involvement of the interprofessional team to manage malnutrition in addition to paediatricians is emphasised. The audiologist’s role is not mentioned.
Udoh et al., 2024 [14]	3.7% (CSOM) (in MAM)	Low socio-economic conditions, overcrowding, and poor hygiene	Diarrhoea, fever, cough, skin/hair changes, ear discharge	The cascading effects of the association between malnutrition and infection are highlighted as a contributor to morbidity in children. No implications or strategies were recommended.

Abbreviations: ENT, ear, nose, and throat specialist; HIV, human immunodeficiency virus; MAM, moderate acute malnutrition; OM, otitis media; PEM, protein-energy malnutrition; SAM, severe acute malnutrition; TB, tuberculosis.

### 3.2. Prevalence of OM in Children with SAM

The reported prevalence of OM in children with SAM ranges from 0.65% to 84.6%. The lowest prevalence was reported by Chaurasiya et al. [32] at 0.65%, followed by Das et al. [26] at 0.79%. Similarly, Sahu et al. [33] reported a prevalence of 2.1%, and K. Das et al. [24] reported 2.5%. Hounkpatin et al. reported a prevalence of SAM in children with OM at 16.3% in their case-control study [3]. Two studies reported a higher prevalence of OM in children with SAM [6,31], at 59.9% and 84.6%, respectively. Comparatively, Udoh et al. [14] found a 3.7% prevalence in young children with moderate acute malnutrition (MAM). Hounkpatin et al. highlight that children with SAM have a 2.23 times higher risk of acute OM compared to well-nourished peers [3].

### 3.3. Risk Factors of SAM and OM

Low socioeconomic status was the predominant risk factor reported, with the majority of children coming from economically disadvantaged families experiencing overcrowding, poor hygiene, limited maternal education, and unemployment [3,14,24,32,34]. Poor feeding practices, including early cessation of exclusive breastfeeding ranging from 10% to 79% across regions and inappropriate complementary feeding, were identified risks [24,26,31]. Biological risk factors were described, including anaemia and other comorbidities, such as TB and HIV [31,33]. Studies report varying male [24,31,32,33] and female [14,26,34] predominance. Environmental factors such as smoke exposure and lack of safe water exacerbate risks, contributing to respiratory infections and recurrent illness [3,6]. Limited parental education [31,34] and low immunisation adherence are also identified as risks [24,26].

### 3.4. Co-Morbidities with SAM and OM

Anaemia was one of the most common co-morbidities, ranging from 20.2% to 84.21% prevalence [24,32,34]. Gastroenteritis (31.75–47.3%), diarrhoea (13.5–56.8%), respiratory tract infections (12.9–46.3%), as well as skin and hair changes (up to 34.92%), associated with SAM, were reported [14,24,26,33,34]. Infections such as bacteraemia, urinary tract infections, malaria, measles, HIV (0.31–1.05%), and TB (0.63–5.6%) were documented [6,24,26,33,34]. Other notable co-morbidities included developmental delay, congenital heart disease, and rarely, meningitis and mastoiditis [6,26,34]. Ear-specific co-morbidities like OM and otorrhoea were reported, as well as conductive and sensorineural hearing loss associated with OM in up to 58% of children with SAM [6,31].

### 3.5. Management Strategies for SAM and OM

Screening weight-for-height measurements and anthropometry for early identification of children at risk of SAM was emphasised [24,32]. Environmental risk identification and mitigation to reduce OM prevalence are highlighted [3]. Community-based intervention and health promotion, such as immunisation and parent education on nutrition, feeding practices, and health monitoring, were advised to prevent OM and SAM [6,24,26,34]. A therapeutic nutritional care program and antimicrobial therapy as well as admission to nutritional rehabilitation centres were shown to effectively improve the clinical outcomes of children with SAM [26,32,33]. Monitoring OM and hearing evaluations for children with malnutrition was recommended [31]. Collaboration between stakeholders, including healthcare practitioners, parents, healthcare facilities, and political and administrative authorities, is promoted for holistic care of children with SAM and OM [3]. Interdisciplinary healthcare, including ENTs, paediatricians, dieticians, and community healthcare workers, was recommended [6,31]. The role of the audiologist was not mentioned.

### 3.6. Research Gaps Identified

Variation in prevalence rates due to methodological inconsistencies and geographic differences indicates the need for further population-based studies to establish prevalence data for OM in children with SAM. Most studies are hospital- or region-specific, leading to differences in OM prevalence in children with SAM being underrepresented across urban, rural, and socio-economically diverse populations. Detailed exploration of how environmental risk factors and parental education interact with SAM and contribute to OM is necessary. Research identifies hearing loss and developmental delays [23,25,26,31,34]; however, longitudinal research should explore the effects of OM in children with SAM. Similarly, research should explore the interaction of SAM and OM with rare co-morbidities such as meningitis or congenital heart disease. Although interprofessional collaboration is recommended [34], there are insufficient guidelines for teams to address OM in children with SAM. It is unclear if the inclusion of hearing screening or evaluations is feasible in existing nutritional programs. Targeted research on otologic health and audiological outcomes of children with SAM is necessary.

## 4. Discussion

This scoping review explored the prevalence, risk factors, co-morbidities, and management strategies of OM in children under five years with SAM. Findings highlight the complexity of biological and environmental factors contributing to SAM and associated OM. Variability in reported rates and gaps in understanding the concepts are highlighted to perpetuate further research and management in this population.

Most participants in the identified studies were between 6 and 24 months old, which is concerning as developmental delays are four times more likely for children in this age range than older children [23]. Health professionals should prioritise nutritional screening for this population to facilitate timely intervention and prevent developmental delays. While the association of OM and malnutrition is well-documented, the prevalence of OM in children with SAM showed wide variation (range: 0.65% to 84.6%). High prevalence is concerning given that OM is a leading cause of morbidity in children globally [6,13]. Studies (n = 5) that reported lower prevalence of less than 5% focused on limited regional and hospital-based populations, which may skew prevalence rates [24,26,32,33,34]. Variance in the prevalence of OM in children with SAM may be attributed to study setting, population characteristics, or methodological differences. Two studies did not include hospital-based populations [3,14]. Specifically, in the included studies, variance across inclusion and diagnostic criteria, such as including acute OM or only chronic or recurrent OM, may change prevalence. The need for further investigation with standardised diagnostic criteria is emphasised to improve understanding of the prevalence of OM in children with SAM.

In congruence with prior systematic reviews, this scoping review identified a complex interplay of biological, environmental, and socioeconomic risk factors for OM and SAM [8,12,20]. Among the identified risk factors, low socioeconomic status was most commonly reported, causing adverse living conditions that contribute to recurrent illnesses [14,32]. Exposure to indoor smoke can contribute to respiratory infections, which can precede and increase episodes of OM [3,35]. Poor feeding practices and early cessation of breastfeeding are associated with limited parental education and undermine nutritional health, compounding risks of OM and SAM [10,18,24]. Studies highlighted biological vulnerabilities such as anaemia, TB, and HIV, which heighten susceptibility to infections such as OM [32,33]. These findings align with the existing literature on SAM and OM, underscoring the cascading effects of risk factors and the compounded vulnerability of children with SAM and OM [4,11,13,14]. Findings highlight the need for further research to prioritise early detection and develop interventions to mitigate risks [3,14]. Health professionals should prioritise screening for the detection of OM and SAM, particularly for children from low socio-economic backgrounds. In alignment with WHO recommendations, integrated healthcare strategies are needed to address biological vulnerabilities, and collaboration for community-based interventions to overcome risk factors is necessary [2,5]. Despite consensus on many aspects, studies show regional and demographic nuances. Differences in male or female predominance and varying rates of exclusive breastfeeding highlight the importance of interventions tailored to regional and community needs [24,26,32].

Children with SAM were frequently reported to have multiple co-morbidities that compromise physiological responses, exacerbate the severity and recurrence of OM, and further complicate OM management [24,32,33]. Children with SAM are at a high risk of OM, particularly children with associated co-morbidities such as HIV and TB, which further compromise immunity and make the ears more susceptible to infections [14,24,33]. SAM and associated co-morbidities can both increase the severity and frequency of recurrent OM infections, necessitating interventions for nutrition, immunity, and infection control [3,14]. Further research is needed to explore the effects of OM comorbidity on the recovery and treatment of children with SAM. A broader understanding of coexisting diseases, particularly OM, in children with SAM could enhance healthcare.

SAM can lead to various auditory complications in children, including OM, moderate-to-profound hearing loss, and high-frequency, sensorineural, or mixed hearing loss [25]. Research identifies children with SAM are at heightened risk of hearing loss, particularly as a complication of OM, highlighting the need for routine hearing evaluations in healthcare for children with SAM [6,25,31]. Early detection and management of OM are critical to mitigate the negative impact on children’s development, particularly speech-language development [7,31].

Children’s immunocompromised state as a result of SAM contributes to a 2.23 times higher risk of acute OM, as demonstrated by Hounkpatin et al. [3,6]. Prior research shows the severity of nutritional deficiency can exacerbate immune dysfunction, increasing the risk of OM, recurrent infection, and potential hearing loss [3,36]. Research shows nutritional interventions can reduce the incidence and severity of infections, including OM, highlighting the need to integrate nutritional strategies to manage auditory health in children with SAM [6]. Audiologists should have an active role in detecting possible hearing loss in children with SAM. There is a need to explore the role of audiologists in managing OM in children with SAM, particularly in complicated SAM cases with HIV and TB co-morbidities. Hearing screenings may be integrated with existing nutritional programs; however, the feasibility of integration and collaboration remains unclear. Auditory impairment in children with SAM frequently relates to damage to the inner ear and auditory neural pathways [36]. Evidence suggests young children with SAM are more vulnerable to the onset and progression of hearing loss, which can be permanent and show limited improvement with nutritional recovery over time [25]. Further research is needed to understand the progression of hearing loss in children with SAM and the potential for hearing recovery to inform protocols and schedules of hearing evaluations.

The findings emphasised that managing OM in children with SAM requires a multifaceted approach. Management strategies identified in this review mostly align with existing WHO guidelines for SAM management [2]. These strategies, including nutritional rehabilitation, early risk identification, infection control, and community-based interventions, should be integrated to sustain recovery and prevent relapse [2,12,26,34]. Consistent with prior research on risk factors for SAM, community-based public health measures to improve hygiene and handwashing, reduce indoor smoke exposure, and educate are promoted to prevent and reduce OM in children with SAM as well [2,3,20,26]. Similar to the WHO guidelines, S. Das et al. and Sahu et al. highlighted antibiotic use to manage infection [2,26,33]. These studies, however, further highlight antibiotic prescription based on the regional resistance profile to prevent antibiotic resistance, reinforcing regional considerations [26,33]. While antibiotic prescription can treat OM in healthy children, few studies evaluate the effectiveness of individual OM treatments in children with SAM. None of the studies informed treatment of CSOM, a severe form of OM that, if left untreated, may cause permanent hearing loss and limit childhood development [32,36].

In alignment with WHO SAM management guidelines [2], research recommends interprofessional approaches to manage OM in children with SAM [34]. The guidelines, however, do not describe the involvement of an audiologist specifically [2]. Although OM and hearing loss can resolve with nutritional recovery, in severe cases such as SAM, recovery may be less, contributing to lifelong hearing impairment and a significant impact on developmental outcomes [25,31]. Despite hearing loss being recognised as a common complication, the role of audiologists was not mentioned in the studies [31,34]. Audiologists can contribute to the early detection of OM, diagnose hearing loss, educate caregivers, and implement prevention strategies. Therefore, this review promotes further exploration of audiological services in SAM management.

### 4.1. Strengths and Limitations

Methodological transparency and adherence to the PRISMA-ScR guidelines are strengths of this review. The process of independent selection and cross-checking selection criteria promoted reliability and accuracy of data extraction. The review findings provide insights into the nature of OM in children with SAM and highlight the need for further exploration to improve health promotion and developmental outcomes for this population. Despite these contributions, the limitations of the review warrant consideration.

Included studies were conducted in developing contexts following cross-sectional, observational designs with primarily hospital-based populations. The generalisability and representation of outpatient populations are thus limited. A significant number of studies (n = 6) were based in India, which introduces geographical bias and can skew findings. Moreover, all studies were from developing contexts, which should be considered when interpreting results. Developing contexts do experience a higher prevalence of SAM [1]. India, in particular, has a very high prevalence of SAM, with 18.7% of children under 5 years wasted [1,36]. Higher research output on SAM in this region is therefore expected. It is important to note that the underlying causes of SAM in developed contexts may be less related to psychosocial aspects and more medical-related, such as dysphagia [37]. Similarly, underlying causes of OM may differ since there is variation in healthcare infrastructure, nutrition, and medical services. As such, findings should be interpreted with caution and may not apply to developed contexts.

As studies in this review were published, publication bias may have influenced findings. Overrepresentation of studies with positive results and underrepresentation of negative or null results may skew conclusions. Future research may consider the inclusion of unpublished data to provide a balanced summary of the topic. A reliability and risk of bias assessment was not completed and is, therefore, recommended for future research. While this review reflects the most recent, relevant evidence base, the timeframe may have excluded historical insights that can improve understanding of OM in children with SAM. If more resources are available for a review, expanding the timeframe and use of consultation can provide a richer dataset in future research. Although the review successfully explored existing evidence, knowledge gaps on OM in children with SAM require further research with longitudinal studies and exploration of the role of the audiologist.

### 4.2. Recommendations for Future Research

Large-scale population-based studies with standardised criteria are needed to quantify the prevalence of OM in children with SAM, considering regional, cultural, and socio-economic variance. Research should use well-defined inclusion criteria such as WHO Sam classification [2] and standardised diagnostic protocols of OM. To enable improved generalisability and validity of research, comparative studies between developed and developing contexts are needed [3,12]. Multi-centre cohort studies across different geographically diverse populations with varying socioeconomic and healthcare environments are recommended. A minimum 500-person sample size per region may enable adequate subgroup analysis.

Differentiating AOM and CSOM in future research is important to establish the unique effects each type has on children with SAM [31]. Case-control studies with stratified types of OM may compare the incidence, risk factors, and progression of OM in children with SAM to inform targeted intervention strategies. Longitudinal studies are needed to understand the nature and dynamic changes of risks in children with SAM over time. Prospective cohort studies involving regular otoscopy, middle ear evaluations, hearing thresholds, and nutritional assessments over 2 years may be effective. A minimum sample size of 200 per cohort may be necessary for robust longitudinal analysis. Further randomised control trials are needed to explore the role of SAM in exacerbating OM. The impact of comorbidities on the incidence, severity, and progression of OM in children with SAM should be explored with cross-sectional studies. Multivariate regression models may account for confounding factors.

Future studies should assess the effectiveness of OM interventions, particularly for CSOM in children with SAM. Cluster randomised control trials may compare antibiotic therapy and audiology services to nutritional rehabilitation to inform interprofessional treatment approaches. Research should involve outcome measures for resolution time, OM incidence, severity, recurrence, and hearing thresholds to identify improvements and progressions. Speech-language assessments may be included as secondary outcome measures. Limited literature is available on the role of audiologists in managing OM in malnourished populations. There is a need for further research on interprofessional approaches to managing hearing and developmental outcomes in children with SAM [33,34,36]. Qualitative research with healthcare practitioners could identify barriers and facilitators to collaboration. Mixed-methods research with 30 to 50 participants per region could inform practice guidelines.

To guide healthcare practitioners, implementation science research is needed to explore the feasibility and risks of integrated nutritional and OM management in children with SAM. A sample of 500 children may effectively explore adherence, cost, and service satisfaction. Addressing these gaps may inform holistic care strategies and improve nutritional, audiological outcomes, and overall health outcomes for children with SAM.

## 5. Conclusions

This scoping review underscores the complex relation of SAM and OM in children under 5 years. Most studies in this review adhered to WHO diagnostic criteria. The review, however, cannot establish a direct relationship between SAM and OM due to limited availability of research identifying SAM according to WHO guidelines and high variability in types of OM described. Future research should follow WHO guidelines for diagnosing malnutrition to further explore the interaction between SAM and OM, particularly in community-based populations.

Significant prevalence of biological and environmental risk factors in children with OM and SAM are identified, which should be addressed through community-based interventions and health promotion strategies to improve health outcomes. The review highlights the critical role of interprofessional collaboration, early detection, and intervention to mitigate the compounded risks of SAM and OM. Community education and involving audiologists can enhance the early detection and management of OM for children with SAM. Regular audiological screening or evaluation and management should be provided to all children with SAM. Integration of hearing screening and evaluations with nutritional programs should be explored as a potential management strategy for this population.

Significant gaps in the literature are highlighted, particularly regarding the role of the audiologist. Prospective research should prioritise large-scale, longitudinal studies with well-defined diagnostic criteria to explore the impact of OM on the developmental outcomes of children with SAM. Addressing the research gaps identified in this review can support comprehensive healthcare strategies to improve the health and audiological outcomes of children with SAM.

## Figures and Tables

**Table 1:** Search terms included.

Nutritional Status	Health Condition	Population
Severe acute malnutrition	Otitis media	Child/Children
SAM	OM	Infant/s
Severe malnutrition	Middle ear infection	Toddlers
Malnutrition	Ear infection	Paediatrics
Malnourished		Childhood
Wasted		
Severely wasted		

Abbreviations: OM, otitis media; SAM, severe acute malnutrition.

**Table 2:** Electronic search strategy.

Concept	Search Terms
Nutritional status	(“severe acute malnutrition” OR “SAM” OR “severe malnutrition” OR “malnutrition” OR “malnourished” OR “wasted” OR “severely wasted”)
Health condition	(“otitis media” OR “OM” OR “middle ear infection” OR “ear infection”)
Population	(“child” OR “children” OR “infant” OR “infants” OR “toddlers” OR “paediatrics” OR “childhood”) Combined nutritional status AND health condition Combined nutritional status AND population Combined nutritional status AND health condition AND population.
Search combinations	

## Data Availability

The data analysed during the review are referenced and presented within the article. Further analysed data are available from the corresponding author on reasonable request.

## Supplementary Materials

The following supporting information can be downloaded at: https://www.mdpi.com/article/10.3390/children12040397/s1, Table S1. Search strategy across databases.
