# Supplementary material for: Otitis Media in Children with Severe Acute Malnutrition: A Scoping Review
Source: Children (Basel). 2025 Mar 21;12(4):397. doi: 10.3390/children12040397 (PMC12025682; doi:10.3390/children12040397)
Supplement: Supplementary file 1 [file children-12-00397-s001.zip › children-3478450-supplementary.pdf]

Otitis Media in Children with Severe Acute Malnutrition: A Scoping Review

Table S1. Search strategy across databases

1. **EBSCOHOST**		
THEME / CATEGORY	SEARCH TERMS (All fields)	Number returned
**Health status measure:**	("severe acute malnutrition" OR "SAM" OR "severe malnutrition" OR "malnutrition" OR "malnourished" OR "severely wasted")	14 157
**Audiological condition:**	("otitis media" OR "OM" OR "middle ear infection" OR "ear infection")	16 833
**Population:**	("children" OR "infants" OR "toddlers" OR "paediatrics" OR "childhood")	125 347
Combined health status measurement AND population.	("severe acute malnutrition" OR "SAM" OR "severe malnutrition" OR "malnutrition" OR "malnourished" OR "severely wasted") AND ("children" OR "infants" OR "toddlers" OR "paediatrics" OR "childhood")	5 645
Combined health status measurement AND audiological condition.	("severe acute malnutrition" OR "SAM" OR "severe malnutrition" OR "malnutrition" OR "malnourished" OR "severely wasted") AND ("otitis media" OR "OM" OR "middle ear infection" OR "ear infection")	684
Combined health status measurement AND audiological condition AND population.	("severe acute malnutrition" OR "SAM" OR "severe malnutrition" OR "malnutrition" OR "malnourished" OR "severely wasted") AND ("otitis media" OR "OM" OR "middle ear infection" OR "ear infection") AND ("children" OR "infants" OR "toddlers" OR "paediatrics" OR "childhood")	**25**
FILTERS APPLIED	Publication range (01/01/2014 – present)<br>Language: English<br>Limit: Full-text	

2. **PUBMED**		
THEME / CATEGORY	SEARCH TERMS (All fields)	Number returned
**Health status measure:**	("severe acute malnutrition" OR "SAM" OR "severe malnutrition" OR "malnutrition" OR "malnourished" OR "severely wasted")	9 303
**Audiological condition:**	("otitis media" OR "OM" OR "middle ear infection" OR "ear infection")	3 835
**Population:**	("children" OR "infants" OR "toddlers" OR "paediatrics" OR "childhood")	536 154
Combined health status measurement AND population.	("severe acute malnutrition" OR "SAM" OR "severe malnutrition" OR "malnutrition" OR "malnourished" OR "severely wasted") AND ("children" OR "infants" OR "toddlers" OR "paediatrics" OR "childhood")	6 946
Combined health status measurement AND audiological condition.	("severe acute malnutrition" OR "SAM" OR "severe malnutrition" OR "malnutrition" OR "malnourished" OR "severely wasted") AND ("otitis media" OR "OM" OR "middle ear infection" OR "ear infection")	27
Combined health status measurement AND audiological condition AND population.	("severe acute malnutrition" OR "SAM" OR "severe malnutrition" OR "malnutrition" OR "malnourished" OR "severely wasted") AND ("otitis media" OR "OM" OR "middle ear infection" OR "ear infection") AND ("children" OR "infants" OR "toddlers" OR "paediatrics" OR "childhood")	**23**
FILTERS APPLIED	Publication range (10 years)<br>Language: English<br>Limit: Full-text	

3. **MEDLINE**		
THEME / CATEGORY	SEARCH TERMS (All fields)	Number returned
**Health status measure:**	("severe acute malnutrition" OR "SAM" OR "severe malnutrition" OR "malnutrition" OR "malnourished" OR "severely wasted")	5 012
**Audiological condition:**	("otitis media" OR "OM" OR "middle ear infection" OR "ear infection")	2 991
**Population:**	("children" OR "infants" OR "toddlers" OR "paediatrics" OR "childhood")	244 485
Combined health status measurement AND population.	("severe acute malnutrition" OR "SAM" OR "severe malnutrition" OR "malnutrition" OR "malnourished" OR "severely wasted") AND ("children" OR "infants" OR "toddlers" OR "paediatrics" OR "childhood")	3 697
Combined health status measurement AND audiological condition.	("severe acute malnutrition" OR "SAM" OR "severe malnutrition" OR "malnutrition" OR "malnourished" OR "severely wasted") AND ("otitis media" OR "OM" OR "middle ear infection" OR "ear infection")	20
Combined health status measurement AND audiological condition AND population.	("severe acute malnutrition" OR "SAM" OR "severe malnutrition" OR "malnutrition" OR "malnourished" OR "severely wasted") AND ("otitis media" OR "OM" OR "middle ear infection" OR "ear infection") AND ("children" OR "infants" OR "toddlers" OR "paediatrics" OR "childhood")	**10**
FILTERS APPLIED	Publication range (01/01/2014 – present)<br>Language: English<br>Limit: Full-text	

4. **SCOPUS**		
THEME / CATEGORY	SEARCH TERMS (Title, abstract, keywords)	Number returned
**Health status measure:**	("severe acute malnutrition" OR "SAM" OR "severe malnutrition" OR "malnutrition" OR "malnourished" OR "severely wasted")	53 913
**Audiological condition:**	("otitis media" OR "OM" OR "middle ear infection" OR "ear infection")	21 573
**Population:**	("children" OR "infants" OR "toddlers" OR "paediatrics" OR "childhood")	1 536 299
Combined health status measurement AND population.	("severe acute malnutrition" OR "SAM" OR "severe malnutrition" OR "malnutrition" OR "malnourished" OR "severely wasted") AND ("children" OR "infants" OR "toddlers" OR "paediatrics" OR "childhood")	16 722
Combined health status measurement AND audiological condition.	("severe acute malnutrition" OR "SAM" OR "severe malnutrition" OR "malnutrition" OR "malnourished" OR "severely wasted") AND ("otitis media" OR "OM" OR "middle ear infection" OR "ear infection")	122
Combined health status measurement AND audiological condition AND population.	("severe acute malnutrition" OR "SAM" OR "severe malnutrition" OR "malnutrition" OR "malnourished" OR "severely wasted") AND ("otitis media" OR "OM" OR "middle ear infection" OR "ear infection") AND ("children" OR "infants" OR "toddlers" OR "paediatrics" OR "childhood")	**87**
FILTERS APPLIED	Publication range (2014 – present)<br>Language: English	

5. **SCIENCE DIRECT**		
THEME / CATEGORY	SEARCH TERMS (All fields)	Number returned
**Health status measure:**	("severe acute malnutrition" OR "SAM" OR "severe malnutrition" OR "malnutrition" OR "severely wasted")	166100
**Audiological condition:**	("otitis media" OR "OM" OR "middle ear infection" OR "ear infection")	112 547
**Population:**	("children" OR "infants" OR "toddlers" OR "paediatrics" OR "childhood")	494 497
Combined health status measurement AND population.	("severe acute malnutrition" OR "SAM" OR "malnutrition" OR "severely wasted") AND ("children" OR "infants" OR "toddlers" OR "paediatrics" OR "childhood")	52 754
Combined health status measurement AND audiological condition.	("severe acute malnutrition" OR "SAM" OR "malnutrition" OR "severely wasted") AND ("otitis media" OR "OM" OR "middle ear infection" OR "ear infection")	3 698
Combined health status measurement AND audiological condition AND population.	("severe acute malnutrition" OR "SAM" OR "malnutrition" OR "malnourished" OR "severely wasted") AND ("otitis media" OR "middle ear infection") AND ("children" OR "paediatrics")<br><br>("severe acute malnutrition" OR "SAM" OR "malnutrition" OR "severely wasted") AND ("otitis media" OR "ear infection") AND ("children" OR "infants")	Total = **717**
FILTERS APPLIED	Publication range (2014 – present)<br>Language: English	

6. **GOOGLE SCHOLAR**		
THEME / CATEGORY	SEARCH TERMS (Anywhere in the article)	Number returned
**Health status measure:**	("severe acute malnutrition" OR "SAM" OR "severe malnutrition" OR "malnutrition" OR "malnourished" OR "severely wasted")	862 000
**Audiological condition:**	("otitis media" OR "OM" OR "middle ear infection" OR "ear infection")	1 150 000
**Population:**	("children" OR "infants" OR "toddlers" OR "paediatrics" OR "childhood")	1 090 000
Combined health status measurement AND population.	("severe acute malnutrition" OR "SAM" OR "severe malnutrition" OR "malnutrition" OR "malnourished" OR "severely wasted") AND ("children" OR "infants" OR "toddlers" OR "paediatrics" OR "childhood")	569 000
Combined health status measurement AND audiological condition.	("severe acute malnutrition" OR "SAM" OR "severe malnutrition" OR "malnutrition" OR "malnourished" OR "severely wasted") AND ("otitis media" OR "OM" OR "middle ear infection" OR "ear infection")	49 200
Combined health status measurement AND audiological condition AND population.	("severe acute malnutrition" OR "SAM" OR "severe malnutrition" OR "malnutrition" OR "malnourished" OR "severely wasted") AND ("otitis media" OR "OM" OR "middle ear infection" OR "ear infection") AND ("children" OR "infants" OR "toddlers" OR "paediatrics" OR "childhood")	21 400<br>**(Used = 100)**
FILTERS APPLIED	Publication range (2014 – 2024)<br>Limit: exclude citations	
